# Supplementary figures and images for: Weekday-Weekend Sedentary Behavior and Recreational Screen Time Patterns in Families with Preschoolers, Schoolchildren, and Adolescents: Cross-Sectional Three Cohort Study
Source: Int J Environ Res Public Health. 2021 Apr 24;18(9):4532. doi: 10.3390/ijerph18094532 (PMC8123206; doi:10.3390/ijerph18094532)

**Scheme S1.** Study flowchart of participants.

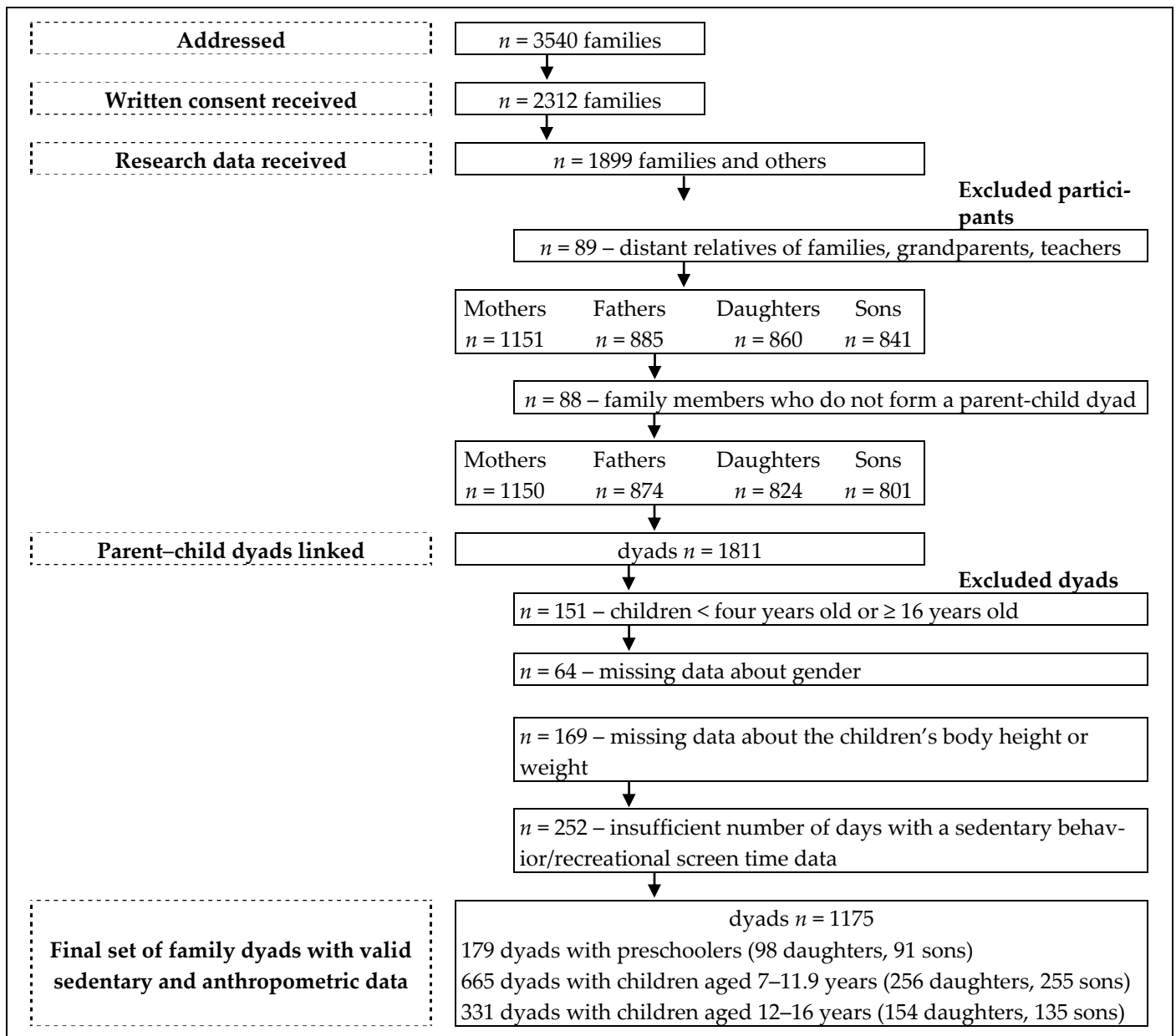

Supplement: Supplementary file 1 [file ijerph-18-04532-s001.zip › ijerph-1169331-supplementary.pdf]
